# Engineered NK Cells Against Cancer and Their Potential Applications Beyond

**DOI:** 10.3389/fimmu.2022.825979

**Published:** 2022-02-15

**Authors:** Maria Karvouni, Marcos Vidal-Manrique, Andreas Lundqvist, Evren Alici

**Affiliations:** ^1^Center for Hematology and Regenerative Medicine, Department of Medicine-Huddinge, Karolinska Institute, Stockholm, Sweden; ^2^Department of Oncology‐Pathology, Karolinska Institute, Stockholm, Sweden

**Keywords:** natural killer cells, chimeric antigen receptors, cancer, autoimmunity, clinical trials, CAR-NK, Preclinical studies

## Abstract

Cell therapy is an innovative therapeutic concept where viable cells are implanted, infused, or grafted into a patient to treat impaired or malignant tissues. The term was first introduced circa the 19^th^ century and has since resulted in multiple breakthroughs in different fields of medicine, such as neurology, cardiology, and oncology. Lately, cell and gene therapy are merging to provide cell products with additional or enhanced properties. In this context, adoptive transfer of genetically modified cytotoxic lymphocytes has emerged as a novel treatment option for cancer patients. To this day, five cell therapy products have been FDA approved, four of which for CD19-positive malignancies and one for B-cell maturation antigen (BCMA)-positive malignancies. These are personalized immunotherapies where patient T cells are engineered to express chimeric antigen receptors (CARs) with the aim to redirect the cells against tumor-specific antigens. CAR-T cell therapies show impressive objective response rates in clinical trials that, in certain instances, may reach up to 80%. However, the life-threatening side effects associated with T cell toxicity and the manufacturing difficulties of developing personalized therapies hamper their widespread use. Recent literature suggests that Natural Killer (NK) cells, may provide a safer alternative and an ‘off-the-shelf’ treatment option thanks to their potent antitumor properties and relatively short lifespan. Here, we will discuss the potential of NK cells in CAR-based therapies focusing on the applications of CAR-NK cells in cancer therapy and beyond.

## NK Cell Biology

Natural killer (NK) cells are characterized as cytotoxic lymphocytes of the innate immune system. They account for 5-15% of the circulating mononuclear lymphocytes and are phenotypically defined as CD3^-^CD56^+^NKp46^+^. NK cells develop in the bone marrow (BM) niche from hematopoietic stem cell (HSC) progenitors and undergo maturation in the BM or other secondary lymphoid organs, such as uterus, liver and tonsils (1). NK cells play important roles in host defense due to their ability to recognize and eradicate viral infected and malignant cells without the need for prior sensitization. They are equipped with a repertoire of receptors responsible for delivering activating or inhibitory signals, the relative balance of which dictates the cytotoxic activity and the canonical functions of the cell, such as proliferation and cytokine release (1, 2). Typical activating receptors are the Natural Cytotoxicity Receptors (NCRs) NKp44 (CD336), NKp46 (CD335), and NKp30 (CD337), as well as the Killer Immunoglobulin-like Receptors (KIRs) KIR2DS1, KIR2DS2, KIR2DS4, KIR2DS5, and KIR3DS1. Other important activating receptors are CD224 (2B4), CD226 (DNAM-1) and NKG2D. Several ligands to these activating receptors are upregulated upon cellular stress, infection, or malignant transformation. In cancer, commonly upregulated ligands include MICA and MICB (MHC-class I polypeptide-related sequence A and B), the UL16-binding proteins (ULBPs) and the adhesion molecules PVR (poliovirus receptor, also known as CD155) and Nectin-2. MICA/B and ULBPs are mediating activating signals *via* binding to NKG2D, whereas PVR and Nectin-2 ligate DNAM-1 (3). NCRs recognize a diverse set of ligands, such as heparan sulfate proteoglycans, cell surface proteins and proteins that reach the surface after their intracellular cleavage. These ligands are not exclusively activating but can also have an inhibitory effect depending on the splice variant of the receptor. The research on the identification of NCR ligands is ongoing. Some of the better studied ones include B7-H6 and HLA-B associated transcript 3 (BAT3) that bind to NKp30, and the proliferating cell nuclear antigen (PCNA) that binds to NKp44. The mechanism of upregulation of NK cell activating ligands is not fully elucidated, although increasing evidence suggests transcriptional and post-translational modifications taking place as a result of cell response to stressful stimuli and DNA damage (4, 5).

Besides the activating KIRs, many of the KIR group receptors are known to propagate inhibitory feedback upon interaction with their ligands. Such ligands are self-MHC (major histocompatibility complex) class I molecules that are expressed in all nucleated cell types and play a critical role in mitigating autoimmune reactions. The downregulation of surface MHC class I can occur under cellular stress conditions leading to increased targeting by NK cells. This is also known as ‘missing-self recognition’ and is a unique feature of NK cells. Inhibitory signals are also mediated by the receptors sialic acid-binding Ig-like lectin-7 (siglec-7) and 9 (siglec-9) that bind to sialic acid-containing carbohydrates (e.g. mucins) aberrantly expressed on tumor cells (6). Other inhibitory receptors are the complex NKG2A/CD94 and the receptors CD161 and KLRG1, that bind to HLA-E, lectin-like transcript 1, and cadherins, respectively.

In addition to the expression of activating and inhibitory receptors, mature NK cell subsets express the FcγRIIIa receptor CD16 that allows the recognition and elimination of antibody-coated cells through antibody-dependent cellular cytotoxicity (ADCC) (7). The mechanism of ADCC is being increasingly explored in cancer therapy. Such therapeutic approaches involve the use of monoclonal antibodies (mAbs) to specifically bind cell surface moieties. Subsequently, CD16- Fc region interaction triggers the antitumor effector immune response resulting in target cell killing. Today, a large number of mAbs have received regulatory approval for cancer treatment, including Rituximab (anti-CD20), Daratumumab (anti-CD38) and Elotuzumab (anti-SLAMF7) (8).

## NK Cell-Mediated Cellular Cytotoxicity

Upon target recognition and immunological synapse formation, NK cells induce target cell lysis *via* the secretion of lytic granules containing perforin, granzymes (mainly granzyme B) and granulysin (9). The process, also known as degranulation, involves the delivery of granzymes into the cytosol of the target cells, through pores formed by perforin. The granzymes are then cleaving several substrates including caspase-3, Bid and DNA-PKc, and initiate target cell death. A second NK cell killing mechanism is mediated by the engagement of the death receptors Fas and TNF-related apoptosis-inducing ligand (TRAIL)-R1/2 expressed on the surface of target cells, to their respective ligands FasL and TRAIL on NK cells (10). This interaction triggers cell death *via* the activation of caspase-8. The two pathways follow different kinetics, as granzyme B mediates killing in shorter time compared to death receptors (11).

Similar to CD8^+^ cytotoxic T cells, NK cells are ‘serial killers’ (12). Observations from time lapse video microscopy revealed that a single NK cell can eliminate up to six target cells. Moreover, it has been shown that NK cells switch from the ‘faster’ granzyme B to the ‘slower’ death receptor–mediated killing during serial target elimination (10). However, the exertion of cytotoxicity, especially when sequential, can lead to depletion of the cytotoxic granule payload, and consequently to NK cell anergy. Strategies to prevent NK cell exhaustion and restore cell fitness have been explored (12). Exposure to cytokines including IL-2, IL-15 and IFNα has been shown to prevent NK cell exhaustion.

Alongside the direct effector cell functions, activated NK cells release major inflammatory cytokines, such as IFNγ, TNFα, GM-CSF, and chemokines, like CCL1-5 and CXCL8 (13, 14). which play important immunomodulatory roles in cellular activation, differentiation, and migration.

## Adoptive NK Cell Therapy

Unlike T cells, the large-scale *ex vivo* expansion of autologous and donor-derived peripheral blood (PB) NK cells has been a challenge for many years. Today, NK cell expansion is performed either by a cytokine-based system or by a feeder cell-based one. The first method includes the use of IL-2, IL-12, IL-15, and IL-21, alone or in a combination of them, to provide the necessary activating and proliferating stimulus (15–17). Alternatively, the stimuli are provided by feeder cells. As feeder cells can be used autologous cells, recombinant human fibronectin fragment-stimulated T cells (18), Epstein-Barr virus-transformed lymphoblastoid cell lines (19), or genetically modified cells of the chronic myelogenous leukemia cell line K562 (20). The latter are engineered to express membrane bound IL-15 (mbIL15), mbIL21, MICA, and/or 4-1BB ligands (21, 22). Of note, feeder cells need to undergo an irradiation step prior to the initiation of the expansion to prevent cell division and obtain high purity of the final NK cell product.

Although an in-depth comparison between the different expansion strategies has yet to be done, it has been shown that the proliferative capacity, the cytotoxic potency (23), the metabolic function (24) and the receptor expression profile of the generated NK cells are heavily influenced by the expansion protocol (21). For instance, NK cell fold expansion can be negatively affected by telomere shortening; a process occurring due to the repeating replication cycles leading to NK cell senescence (25). The degree of telomere shortening is evidenced to be affected by the protocol used. Denman and colleagues showed that NK cell expansion with mbIL21-expressing feeder cells sustained or even increased telomere length while inducing a mean of 47,967-fold expansion (22). In comparison, the NK cell expansion with mbIL15-expressing feeder cells was limited to 825-fold and telomere shortening was observed (22, 26). Regarding the phenotype of the *ex vivo* expanded cells, studies have focused on the effect of the expansion in the upregulation of immune checkpoints and immunosuppressive molecules, as this can indicate limited efficacy. Markers associated with T cell exhaustion, such as PD-1 and Tim3, have been indeed found upregulated in healthy donor NK cells expanded with a clinically validated protocol of mbIL15-mb4-1BBL-K562 feeder cells + soluble IL-2 (27). Nevertheless, *in vitro* responsiveness assays verified the high cytotoxicity of the cells, suggesting that despite the upregulation of these markers, the cells were not functionally exhausted. Similar conclusions were drawn from cytokine-based NK cell expansions (28). Today, an increasing number of clinical trials is choosing feeder cell expansion systems. However, feeder-free expansion is still a feasible option as it is easier to adapt to GMP regulations and does not involve the hazard of infusing viable feeder cells to the patients (29).

In 2011, Parkhust and colleagues reported the infusion of *ex vivo* cytokine stimulated PB-derived autologous NK cells (30). Although the infusion was well-tolerated, none of the eight recruited patients responded to the treatment. This was hypothesized to be due to the inhibitory interactions between the NK cells and self MHC-class I molecules that are upregulated within the tumor microenvironment (TME). Moreover, the patients were heavily pre-treated, which by itself could have a negative impact on the function of NK cells. Nonetheless, the study provided valuable insight on the persistence of the cells *in vivo* reporting the presence of NK cells in peripheral blood of the patients between a week and several months post infusion. In an attempt to improve NK cell targeting of tumors, Lundqvist and colleagues investigated pre-treatment with the proteasome inhibitor Bortezomib (31, 32). Infusions of *ex vivo* expanded NK were well-tolerated with the exception of thyroiditis and constitutional symptoms related to IL-2 therapy. The study showed preliminary clinical evidence of antitumor immunity with best clinical response observed in 7/14 patients having stable disease (33).

In comparison to autologous NK cells, NK cells from haploidentical donors can elicit greater cytotoxicity due to the alloreactivity caused by the KIR-HLA mismatch (34, 35). This observation was made in an acute myeloid leukemia (AML) mouse model transplanted with HSC, and its translation to the clinic provided grounds to investigate haploidentical or HLA-mismatched NK cells in adoptive cell transfers (36). In 2005, Miller and colleagues conducted a phase I clinical trial in patients with poor-prognosis AML (37). Results showed that infusions of alloreactive NK cells derived from haploidentical donors are safe, have better *in vivo* persistence and resulted in remission of 5 out of 19 patients. Overall, the fact that allogeneic NK cells have low risk of causing graft-versus-host disease increased the applicability of NK cell therapy and encouraged discussions on off-the-shelf cell products (38).

## Alternative NK Cell Sources

To date, the majority of clinical studies on NK cell adoptive cell therapy (ACT) has utilized NK cells derived from peripheral blood. Through the years other NK cells sources have been explored (See Table 1). An example is the umbilical cord blood (UCB). UCB is a NK cell-rich source readily available as cryopreserved biobank material (39). The high proliferative capacity of the cells is particularly attractive, since it allows the generation of large amounts of clinical-grade NK cells (40). Furthermore, UCB-NK cells are suitable candidates for genetic manipulation strategies (41), as well as for combinational treatments with monoclonal antibodies (40), which allows their applicability in different immunotherapeutic strategies. A limitation of UCB-derived NK cells is the inevitable heterogeneity between the final NK cell products due to the use of different UCB donors among batches (42). In addition, comparative studies between PB NK and UCB-NK showed that the latter have some immature characteristics and phenotypic differences. More specifically, UCB-NK cells have increased expression of NKG2A and decreased expression of CD16, KIRs, target adhesion molecules (CD2, CD11a, CD18 and CD62L), perforin and granzyme B (43, 44). Strategies to overcome these issues include the culture of UCB-NK cells with the EBV-transformed HLA-I^+^ B lymphoblastoid cell line PLH, which provides the necessary inhibitory and activating signals to drive their maturation (45). Moreover, UCB-NK cells are often combined with cytokine support (e.g., IL-2 or IL-15) that enhances their *in vivo* activity and persistence. Nevertheless, it is worthy of mention that a direct comparison between genetically modified PB and UCB-NK led by Herrera and colleagues found both cell sources to induce similar levels of targeted *in vitro* cytotoxicity (46).

**Table 1:** Advantages and limitations of NK cell sources.

NK Cell Source	Advantages	Limitations
*Peripheral blood*	Easy collection; Safe; High cytotoxic potency	Time consuming and costly expansion process; Low numbers in patients; Variability between the final products
*Umbilical cord blood*	Readily available; Safe; High starting percentage of NK cells; Strong proliferation potential	Small volume of starting material; Diverse products depending on the UCB unit; Need of cytokine support for adequate cytotoxic function
*Induced pluripotent stem cells*	Easy generation of high NK cell numbers; Homogenous product; High cytotoxic potency	Additional step of generating NK cells from iPSCs; High production cost
*NK cell lines*	Accessible; Easy to culture and amplify; Fast proof-of-concept studies	Safety concerns; Potential decreased cytotoxicity due to the necessary irradiation step

Stem cell-derived NK cells have been proposed as a viable alternative due to their suitability in standardized off-the-shelf settings. Different sources of stem cells have been explored so far, such as human embryonic stem cells (hESCs) (47), CD34^+^ HSCs (48) and induced pluripotent stem cells (iPSCs) (49). iPSCs have the advantage of being easier to generate and satisfy the clinical interest of providing greater donor diversity regarding KIR haplotypes (47). It has also been shown that irrespective of their KIR expression profiles, iPSC-NKs have similar killing capacity between them (50). iPSCs were firstly reported in 2006, when human somatic cells were reprogrammed by the simultaneous introduction of four factors: *OCT3/4, SOX2, c-Myc and Klf4 (*51, 52). In 2007, the combination of the factors *OCT4, SOX2, NANOG* and *LIN28* was also proved effective (53). After successful reprogramming, iPSCs can undergo an essentially indefinite expansion *in vitro* and, subsequently, produce unlimited amounts of NK cells (54). Their production method is well described (42, 55, 56). Briefly, TrypLE-adapted iPSCs are cultured with human stem cell (SCF) and vascular endothelial growth (VEGF) factors for one week to induce their hematopoietic differentiation. Cells are then further differentiated into NK with the addition of IL-3, IL-15, IL-7, SCF and ftl3 ligand and expanded using feeder cell systems. Similar to UCB-NK cells, iPSC-NK are well susceptible to genetic manipulation and exhibit potent cytotoxicity (57). However, in comparison, the iPSC-derived NK cell population is reproducibly homogenous and consistent (58). Phenotypically, iPSC-NK cells have many similarities to the PB NK cells, with the exception of higher NKG2A and lower KIR expression, which indicates a degree of immaturity (49). The popularity of iPSC-NK cells has been increasing over the past years, with multiple preclinical studies and one clinical trial underway (See **Table 2**).

**Table 2:** Ongoing clinical trials using genetically modified NK cells.

Target	Disease	NK cell source	Intracellular domains	Clinical stage	NCT number
CD7	Lymphoma and leukemia	NK-92	CD28 + 4-1BB + CD3ζ	I/II	NCT02742727
CD19	Acute lymphocytic leukemia, Chronic lymphocytic leukemia, Follicular Lymphoma, Mantle Cell Lymphoma, B-cell Prolymphocytic Leukemia, Diffuse Large Cell Lymphoma	NK-92	CD28 + 4-1BB + CD3ζ	I/II	NCT02892695
CD19	B cell lymphoma or leukemia	UCB-NK	CD28 + CD3ζ	I/II	NCT03056339
CD19	B cell lymphoma	NK-92	2B4	I	NCT03690310
CD19	Non-Hodgkin lymphoma	Not known	Not known	I	NCT04639739
CD19	Non-Hodgkin lymphoma	Not known	Not known	I	NCT04887012
CD19	Non-Hodgkin lymphoma, Chronic lymphocytic leukemia and B cell acute lymphocytic leukemiaB-ALL	Allogeneic NK	Not known	I	NCT05020678
CD19	B cell lymphoma	UCB-NK	Not known	I	NCT04796675
CD19	B cell lymphoma, Chronic lymphocytic leukemia	iPSC	NKG2D + 2B4 + CD3ζ	I	NCT04245722
CD19	B cell lymphoma, Myelodysplastic syndrome	UCB-NK	Not known	I/II	NCT05092451
CD19/CD22	B cell lymphoma	Not known	2B4	I	NCT03824964
CD22	B cell lymphoma	Not known	2B4	I	NCT03692767
CD33	Acute myeloid leukemia	Not known	N Not known	I	NCT05008575
CD33	Acute myeloid leukemia	NK-92	CD28 + 4-1BB + CD3ζ	I/II	NCT02944162
BCMA	Multiple Myeloma	NK-92	4-1BB + CD3ζ	I/II	NCT03940833
BCMA	Multiple Myeloma	UCB-NK	Not known	I	NCT05008536
HER2	Glioblastoma	NK-92	CD28 + CD3ζ	I	NCT03383978
Mesothelin	Epithelial Ovarian Cancer	PB-NK	2B4	I	NCT03692637
MUC1	Hepatocellular carcinoma, Non-small cell lung cancer, Pancreatic carcinoma, Breast cancer, Glioma of brain, Colorectal carcinoma, Gastric carcinoma.	NK-92	CD28 + 4-1BB + CD3ζ	I/II	NCT02839954
NKG2DL	Solid tumors	PB-NK	Not known	I	NCT03415100
NKG2DL	Acute myeloid leukemia, Myelodysplastic syndrome	Allogeneic NK	Not known	I	NCT04623944
NKG2DL and/or SARS-CoV-2 S protein	COVID-19	UCB-NK	Not known	I/II	NCT04324996
PD-L1	Head and neck squamous cell carcinoma, gastric cancer	NK-92	Not known	II	NCT04847466
PSMA	Castration-resistant prostate cancer	NK-92	2B4	I	NCT03692663
ROBO1	ROBO1^+^ solid tumors	NK-92	4-1BB + CD3ζ	I/II	NCT03940820
ROBO1	Pancreatic Cancer	NK-92	4-1BB + CD3ζ	I/II	NCT03941457
ROBO1	Pancreatic cancer	NK-92	4-1BB + CD3ζ	I/II	NCT03931720
–	Non-small cell lung cancer	NK-92	Not known	I	NCT03656705

A newer addition to the NK cell sources has been the memory-like (ML) NK cells. ML-NK cells are generated after viral infection (59), exposure to haptens (60) or cytokines, such as IL-12, IL-15 and IL-18 (61). These cells exhibit characteristics of adaptive immunity and have been reported to have higher anti-cancer reactivity in a clinical setting compared to conventional NK cells (62). The cells are well-susceptible to genetic manipulation and have been recently investigated in the context of chimeric antigen receptor (CAR)-NK therapy where introduction of an antiCD19-CAR further improved their targeting and cytotoxic activity (63).

In addition to primary NK cells, NK cell lines have also been used in ACT. Cell lines have the advantage of being easy to culture, expand and cryopreserve. Out of the available ones, NK-92, an IL-2 dependent non-Hodgkin lymphoma NK cell line, has been tested the most in proof-of-concept, preclinical and clinical settings. NK-92 cells have characteristics of activated NK cells, while lacking KIR expression (except KIR2DL4), which explains the potent cytotoxic responses they exhibit upon target cell recognition (64). The cell line NK-92, as well as its genetically modified IL-2- independent counterpart NK-92MI, have been considered for the use in ‘off-the-shelf’ NK cell-based immunotherapies. KHYG-1 and YT are two other NK cell lines with great potential (65). Nevertheless, the use of transformed cell lines in patients is in general met with safety concerns regarding the tumorigenic nature of the cell lines and the hazard of causing secondary NK lymphoma to the patients (66). These limitations can be overcome by implementing a high-dose (5-10 Gy) γ-irradiation step prior to the cell infusion (67, 68). While irradiation halts cell division, it can also negatively impact their long-term *in vivo* persistence which is necessary for better tumor control (69). Indeed, a study of irradiated and non-irradiated CAR-NK-92MI cells, demonstrated that irradiation with 5Gy reduced *in vitro* and *in vivo* proliferation of the cells and shortened their life span (69). Still, the cytotoxicity against target cell lines is not significantly compromised as shown by two different studies (69, 70). Another concern of γ-irradiation is the cellular damage that it causes, ranging from DNA breakage to radical formation and impairment of the cell membrane integrity (71, 72). An alternative method to γ-irradiation was proposed by Walcher and colleagues. Specifically, they demonstrated that low energy electron irradiation has considerable advantages, as it requires shorter treatment times, has more reproducible dose rates, is easier to implement in a laboratory or GMP setting and -importantly- maintains the high cytotoxic effector function of the NK-92 cells (73).

The unique advantages and weaknesses of each NK cell source are listed in **Table 1**.

## Cryopreservation of NK Cells

The great potential of NK cells as off-the-shelf cellular treatments is often overshadowed by their sensitivity to cryopreservation. For instance, it has been shown that although cryopreserved NK cells can eliminate target cells in standard *in vitro* cytotoxicity assays, their efficacy against three-dimensional tumor models is reduced due to a 6-fold decrease in motility (74, 75). Moreover, in a mouse model where infusion of fresh or cryopreserved expanded NK cells was compared, the disadvantage of cryopreserved cells in homing, persistence and expansion was evident (76). Maintaining NK cell viability and cytotoxic function post-thaw is essential in a clinical setting, where high consistency and quality must be guaranteed. A number of studies are investigating the optimal cryopreservation conditions, assessing various freezing/thawing media, cooling rates, storing conditions, culture protocols and resting times. Although a gold standard has not been established yet, it is worth mentioning that substituting dimethyl sulfoxide (DMSO) with other small molecule sugar or protein-based cryoprotectants (e.g sucrose, proline, mannitol) in the freezing media formulation is a generally accepted alternative, associated with improved viability of immune cells post-thaw (77). Given the fact that cryostoring is a necessary step between the development of a cellular product and its infusion, intensifying the efforts to optimize the procedures will positively impact the universal application of immunotherapy.

## Genetic Manipulation of NK Cells

In the clinical setting, the potency of ACT is dependent on the persistence of the infused NK cells. Accumulation of inhibitory factors, insufficient tumor targeting and failure to persist and expand *in vivo* are only a few of the challenges that need to be overcome. For most of these issues, genetic manipulation of the cell product provides a viable solution. NK cells, however, are typically less susceptible to such manipulation compared to other immune cell types (78). Therefore, although the genetic reprogramming of NK cells shares similar principles and methodologies to that of T cells, additional steps are often necessary.

Transfection is a method in which plasmid DNA, mRNA or proteins are introduced to a cell with the aim to initiate their expression. Depending on whether a short or a long-term expression is desired, different methods are applied. One of the most common transient transfection techniques is electroporation. The method uses electric pulses to permeabilize the cell membrane and create pores from where the genetic material is inserted. Electroporation is highly efficient in T cells, however, similar yields have yet to be observed in NK cells (79). Moreover, electroporation results in short-term expression of the transgene which can limit its applicability in the context of immunotherapy (80). Still, the cost-effectiveness of the technique and the ease of its application in large-scale clinical settings are attractive. Efforts to improve NK cell transfection efficiency focus on optimizing fundamental parameters, such as the number of cells, the voltage, and the concentration of material to be electroporated (81). Moreover, ways to minimize the considerable cell death that follows electroporation are being explored (82). Examples of the application of the method in NK cell therapy are mostly concerning CRISPR-mediated genome engineering (knock outs or edits) (83). If long-term expression is desired, the transposon-based technology may be an attractive method. Two such systems have been reported, namely the Sleeping Beauty (84) and the PiggyBac (85). These systems combine the efficiency of electroporation with the precise insertion of the genetic material into the host genome thanks to its integrating element. Nonetheless, to this day, the use of transposon-based systems is more commonly used in T cell rather than in NK cell studies.

Viral transduction is one of the most common methods for immune cell engineering. It results in stable transgene expression while maintaining higher cell viability compared to electroporation. Moreover, the method is well validated in preclinical and clinical studies, where it has repeatedly proven safe and efficient. The vast majority of the clinical studies are using either lentiviral (LV) or retroviral (RV) vectors, although some studies with adenoviral vectors have also been completed with mixed results (86). The first report of viral transduction of NK cells was made by Nagashima et al., in 1998, where he described the transduction of NK-92 cells using RVs (87). This study showed a transduction efficacy of about 2-3%, which although impressive for the time, it is considered low with today’s standards. Of note, recent publications of retroviral transductions of expanded NK cells resulted in about 70% transduction efficiency (88–90). RVs, and more specifically, self-inactivating γ-RVs were also the first viral vectors to enter clinical trials. Their application was further increased by the development of clinical grade RV-producing packaging cell lines. These cell lines, such as the murine cell line PG-13, are able to continuously generate large quantities of clinical-grade virus supernatant following their stable transduction by the vector of interest (91). A disadvantage of the RVs is the fact that they require active cell division in order to successfully integrate their vector to the host genome (92). Moreover, the integration itself can be at random sites, which rises concerns on tumorigenicity and the overall safety of the approach. Careful optimization of the transduction process is, therefore, necessary in order to limit the viral copy number per cell to the absolute minimum. Apart from the γ-RVs that have been monopolizing the transductions, α-RVs are gaining more attention, especially after studies showing superior transduction efficacies in primary NK cells of α-RVs compared to γ-RVs and LVs (93).

Unlike the RVs, LVs are able transduce cells irrespective of their cell cycle phase, leading to theoretically higher transduction efficacies. The vector of choice for T cell-based immunotherapies is the vesicular stomatitis virus (VSV-G) pseudetotyped vector, because of its broad tropism. However, these vectors have proved less effective in NK cells. Alternatively, LVs pseudotyped with a modified baboon envelope glycoprotein (BaEV-LVs) hold greater potential since their entry receptors sodium-dependent neutral amino acid transporter-1 and -2 (ASCT-1 and ASCT-2) are abundant in NK cells (94). Indeed, two independent investigations showed a 20-fold higher transduction efficacy with BaEV-LVs compared to VSV-G-LVs (94, 95). As far as safety is concerned, LVs, and more specifically the 3^rd^ generation vectors, are considered safer viral vector options since the packaging genes *gag/pol* and *rev* are found in separate plasmids, thus making the generation of wild-type recombinant virus harder (96).

Due to their innate antiviral defense mechanisms, viral transduction of NK cells has proved to be challenging. A way to enhance the transduction efficacy is by reducing the NK cell virus repulsion, which in turn increases the internalization of viral particles into the cells independently of the viral receptors (97). Examples of such reagents are the cationic polymers polybrene and protamine sulfate. Although both reagents are routinely used in viral transduction protocols, they are also associated with decreased post-transduction cell viability. An alternative approach is to use recombinant human fibronectin fragment such as RetroNectin. RetroNectin is a 63kDa with adhesion sites for cells (integrin receptors VLA-4 and VLA-5) and viral particles (heparin domain) (98). Upon adhesion of both parties to the filament, transduction is facilitated thanks to the close proximity between them (99, 100). Transduction using RetroNectin is efficient and suitable for difficult to transduce or frail cells (98). Understanding the intracellular patterns of foreign genomic material recognition has contributed to finding additional strategies to increase transduction efficacy. More specifically, the inhibition of the TBK1/IKKϵ complex acting downstream of RIG-1, MDA-5 and TLR3, has significantly improved the viral transduction (101). Such reagent is the VyOz, which is reported to increase the efficacy of NK cell transduction by 4-fold (102). Of note, all the afore-mentioned reagents are available in GMP grade, further enabling the translation of the preclinical studies to the clinical setting.

Virus-like particles have also recently emerged as gene editing tools. One of these approaches uses engineered murine leukemia virus-like particles loaded with Cas9-sgRNA complexes (103). The system, commonly known as Nanoblade, is suitable for both *in vitro* and *in vivo* manipulations, providing high gene editing efficacy and precision in a cost-effective way. Although they have yet to be applied to NK cells, nanoblades have shown promising results in genome editing of human T, B and CD34^+^ cells (104).

## Chimeric Antigen Receptors (CARs)

Irrespective of the source of NK cells, the cytotoxic potential and the targeting capacity of the cells can be further increased with the expression of CARs. CARs are synthetic receptors comprising of three main regions; the antigen-binding single chain variable fragment (scFv), a short transmembrane region (TM) and one or more signal transduction domains (105).

### Single Chain Variant Fragment (scFv)

The extracellular part, or ectodomain, derives from a tumor-specific antibody and consists of a heavy and a light chain that connect *via* a linker. Selecting the right targeted epitope, scFv and linker for each application is crucial. Indeed, a study comparing distinct scFvs targeting the same antigen demonstrated that the scFv domain can influence the expression of the CAR, as well as its functionality (106). A step further is the affinity optimization of the scFv. This is particularly relevant in the occasions where the targeted antigen is expressed on normal tissues (although in lower levels), and thus there are increased chances of on-target off-tumor toxicities. A strategy to reduce the recognition of antigen^low^ normal cells was described by Drent et al. (107). The researchers used ‘light-chain exchange technology’ to construct 124 new antibodies with 10 to >1000-fold less affinity to CD38, a target for multiple myeloma (MM). The selected scFvs were then assessed in both CAR-T (107) and CAR-NK (65) models, where effector cells expressing the affinity optimized CARs effectively discriminated between MM and normal cells. The selection of the scFv domain can be aided by modern computational methods of protein design (108).

### Hinge Region

The ectodomain is connected to the TM *via* the spacer or Hinge region. Similar to the linker, the type and length of the Hinge region can influence the binding capacity of the CAR, as it provides stability and flexibility to the receptor (109). The right selection of Hinge region can additionally protect the patient from off-target activation of the engineered cells and/or unintentional innate immune response; a phenomenon firstly observed in CARs with an immunoglobulin G (IgG) 1 Fc spacer domains interacting with IgG FcγRI receptors (110). It has also been reported that incorporation of a Hinge region to the CAR design enhances the expansion of CAR-transduced T cells (111). The most commonly used Hinge regions in CAR-NK cell therapy derive from the CD8a, CD28 and the IgG-based sequences (105). To our knowledge, there is no study comparing the Hinge regions in CAR-NK cells. Nonetheless, a study comparing the human CD28 and CD8a regions in anti-CD19 CAR-T cells showed that CD8a induced lower production of IFNγ and conferred better resistance to activation-induced cell death, while maintaining equal cytotoxicity to the CD28 counterpart (112). Apart from the functional purpose, Hinge regions act as a target for CAR-detection antibodies facilitating the confirmation of CAR expression (111).

### Transmembrane Region

After the Hinge region follows the TM. The TM region is responsible for anchoring the receptor on the cell membrane, as well as for transducing the signal from the extracellular to the intracellular domains of the CAR. Typical TMs used in T and NK cell studies are derived from CD3, CD8α and CD28 (112). In the context of NK cell therapy, TM domains of activating receptors, such as CD16, NKp44, NKp46, NKG2D, 2B4 and DNAM-1, have also been tested in an *in vitro* iPSC-NK based study (113). The comparison revealed that the combination of NKG2D-derived TM with 2B4 co-stimulatory domain and CD3ζ signaling domain confers strong antigen-specific cytotoxicity.

### Intracellular Domains

Regarding the intracellular region, the design of the CARs has advanced throughout the years, from having only a signal transduction domain (1^st^ generation CAR) (114), to having one or two co-stimulatory domains additionally (2^nd^ and 3^rth^ generation respectively) (115, 116). The intracellular domains for the first CAR designs were inspired by the activating signaling pathways of T cells. To this day, the most broadly used co-stimulatory domains are CD28 and 4-1BB (CD137), while the most common signal transduction module is CD3ζ (117). Both CD28 and 4-1BB are shown to recapitulate natural co-stimulation and provide increased potency to the transduced effector cells. It is also known that CD28 confers a different set of advantages over 4-1BB (118). Studies comparing the two in a 2^nd^ generation CAR setting showed that 4-1BB promotes survival and proliferation, whereas CD28 attributes a stronger cytotoxic potential. Depending on the clinical application, the use of either or both domains is preferred.

Transitioning into NK cells, the expression of already validated T cell-based CAR designs was initially assessed. The first CAR-NK cell was reported by Tran et al. (119). In this *in vitro* study NK cells successfully expressed a functional CD4-CD3ζ CAR, which redirected the cells against HIV-infected CD4^+^ T cells and NK cell-resistant tumor cells. Soon after, applying the CAR know-how from T to NK cells without adjustments was deemed sub-optimal. This is because besides some common signaling moieties that the two cell types share, such as CD3ζ and 4-1BB, most of the frequently used co-stimulatory domains are absent in NK cells. Therefore, questions were raised on whether the true potential of CAR-NK therapy was harnessed with the early CAR designs. Novel constructs for NK cell therapy are substituting the signaling domains of the CARs with those of activating NK cell moieties. The domains that NK cells typically use for downstream signaling are the CD3ζ, DAP10, DAP12 and FcRγ chains (120, 121). In comparison to CD3ζ that has three ITAM domains, the rest of the molecules have a single ITAM. It is also worth mentioning that DAP10 is the adapter protein of NKG2D, whereas DAP12 mediates signaling of activating KIRs, NKG2C and NKp44. In experiments of primary NK cells, DAP10 was found to be functional only when NKG2D was used as the ectodomain, whereas when NKG2D was utilized as the TM region, DAP10 decreased the functionality of the CAR (122, 123). On the contrary, DAP12, as a signaling domain, was met with greater success outperforming CD3ζ-based CARs in two independent *in vitro* and *in vivo* studies of primary NK cells (124, 125). Another co-stimulatory domain under investigation is the NK cell specific receptor 2B4. A couple of studies comparing NK-92 and PB-NK cells expressing 2^nd^ generation antiCD5 or antiCD123 CAR constructs with either 2B4 or 4-1BB co-stimulatory domains, showed that 2B4-CD3ζ CARs provided superior antitumor efficacy compared to 4-1BB-based CAR constructs (121, 126). A similar effect was observed in a study comparing NK-like CARs (NKG2D TM and 2B4 + CD3ζ intracellular domains) and T-like CARs (CD28 TM and 4-1BB + CD3ζ intracellular domains), which showed that iPSC-derived NK cells expressing NK-like CARs induced tumor regression and prolonged mice survival (113). NK cell-like stimulatory domains are being increasingly used in the design of CAR constructs for NK cell immunotherapies, especially for the treatment of solid tumors (113, 125, 127, 128). Regarding 3^rd^ generation CARs, the investigation on finding the optimal combination of signaling domains is currently of high interest. Overall, the findings suggest that NK cell-like co-stimulatory domains unleash superior antitumor responses by CAR-NK cell products.

## Advancements in CAR and Transgene Design

### Alternative Antigen Recognition Domains

The first CAR designs were comprising of scFv domains deriving from mouse antibodies. It has been shown, however, that murine scFvs can be immunogenic and that the triggering of the host immune response often results in early elimination of the CAR products (129). In an effort to prevent that, fully human CARs are being developed, showing comparable targeting capacity to the originally reported scFvs (130). Besides scFvs, which are typically used as the antigen recognition domain, alternatives for improving the CAR features or facilitating their design process have emerged throughout the years. For instance, when target cells express a cell-specific surface antigen whose ligand is known, CAR constructs can incorporate the ligand itself as the extracellular antigen-recognition domain, instead of an scFv. Of note, Zhuang and colleagues demonstrated that CD28H-based CAR-NK cells could be used to recognize and kill target cells expressing B7H7, its natural ligand (131).

A more recent advancement is the use of nanobody-based constructs. Nanobodies, also known as V_H_H antibodies, derive from the variable domain of heavy chain-only antibodies naturally existing in Camelidae and shark species (132, 133). Nanobodies have several advantages over traditional antibodies, such as increased capacity of reaching inaccessible epitopes thanks to their long CDR3 sequence, ability to maintain their physiochemical properties in extreme conditions, easier humanization process and less probable folding and assembly issues (134–136). A study of generating CD7-nanobody based CAR-NK-92MI cells demonstrated potent antitumor effect against T-cell leukemia cell lines and primary cells (137). Similarly, nanobody-based CAR-NK-92 cells targeting CD38 in multiple myeloma showed high specificity and cytotoxic activity in primary human bone marrow samples (138). A comparison between scFv- and nanobody- based CARs was done in clinical studies of CAR-T cells, where comparable efficacy and safety were reported (136). A further step is the design of affinity optimized nanobodies, using a recently developed algorithm that helps predict the residues whose modulation would confer specific binding characteristics (139).

### Ectopic Cytokine Production

As previously mentioned, the long-term persistence of NK cells after adoptive cell transfer can be a concern without cytokine support (21). Due to the association of cytokine administration with serious adverse effects, recent efforts are incorporating an ectopic cytokine support system into the CAR plasmid, developing thus a so-called ‘armored’ CAR. In a study of Liu et al, CB-NK cells were transduced with a viral vector encoding for an anti-CD19 CAR and the IL-15 gene (41). The results were promising as the generated NK cells exhibited increased cytotoxicity against CD19-positive targets *in vitro* and led to prolonged survival *in vivo (*41). In a phase I/II clinical trial, the same approach showed response to 8/11 patients, without the cause of serious adverse effects (140). In a different study, Wang et al. coupled IL-15 transgene expression to an inducible MyD88/CD40 system and achieved increase of the *in vivo* CAR-NK cell persistence for a minimal of 40-50 days (141).

### Safety Switches

Ensuring the safety of the adoptive cell transfer is pivotal, especially after the reports of neurotoxicity, on target-off tumor effects and cytokine storm in CAR-T cell clinical studies (142). Although NK cells have in general a safer profile than T cells, additional safety measures can be lifesaving during an emergency situation. A method to rapidly terminate a cell-based treatment is by incorporating a suicide gene into the therapeutic transgene. In NK cells, the strategy has been tested using the inducible safety switch caspase-9 (iCasp9) suicide gene that expresses a modified caspase-9 fused to the human FK605 binding protein (41). The system is pharmacologically activated, meaning that upon administration of AP1903 (a chemical inducer of dimerization) a caspase-mediated apoptosis is induced (143). Similar drug-induced CAR-NK cell elimination was achieved by an orthogonal rapamycin-regulated caspase-9 switch (141). Notably, cell-cycle dependent suicide genes, such as HSV-TK, are not recommended for use in CAR therapy, as they require active cell division in order to function and can, additionally, induce immunogenicity (144). An alternative strategy for increasing safety and minimizing on-target off-tumor effects was applied in an anti-CD147 CAR-NK study for hepatocellular carcinoma (70). Tseng and colleagues designed a GPC3-synNotch-inducible anti-CD147 CAR, in which the CAR-mediated antitumor responses were unleashed only when GPC3 and CD147 were co-expressed on the surface of the target cell.

### Dual-Specificity CARs

Antigen escape, the partial or total loss of the targeted antigen expression from the surface of malignant cells, is a known mechanism that impedes the efficacy of CAR therapy in cancer. A study addressed this issue by designing a CAR construct with dual specificity for the tumor associated antigens (TAA) EGFR and its mutant form EGFRvIII for the treatment of glioblastoma (145). Dual-specific NK cells eliminated cells positive for both or either of the antigens, in contrast to the CAR-NK cells targeting a single epitope, which resulted in the significant extension of survival of glioblastoma bearing mice. A different approach was proposed by Li and colleagues, who engineered a plasmid encoding for NKG2D and an antiPD-1-CAR, with the intracellular domain of DAP10, aiming to induce synergistic activation of NK-92 cells by parallel recognition of PD-1 and NKG2D ligands (146). Functional assays showed increased cytotoxic activity of the engineered cells and underlined the potential of the method. Notably, dual targeting can be also achieved with the use of tandem CAR constructs. Tandem CARs are constructed by consecutively linking two different antigen binding domains (either scFv or nanobody-based) to a single intracellular domain. Although this approach has been assessed in CAR-T studies, tandem CARs have not yet been evaluated in the NK cell setting (147).

### Adapter CARs

Grote S. et al. further increased the versatility of CAR-targeting by proposing modular CARs (148). Modular or ‘adapter’ CARs (AdCARs) recognize biotin-labeled antibodies specifically targeted against TAAs. This would mean that the CAR-mediated cytotoxicity is fully dependent on the selection of the biotin-labeled antibodies and can be easily modified if needed. The novel AdCAR-NK-92 cell product demonstrated superior cytotoxic responses against CD19^+^ and/or CD20^+^ primary cell targets and gave ground for discussions on off-the-shelf universal CAR-NK cell therapy.

## Approaches to Modulate CAR-NK Cell Functionality

### Expression of Chemokine Receptors and Cytotoxic Ligands

Cell therapy is traditionally administrated *via* intravenous infusion. Therefore, trafficking of CAR-NK cells to the targeted tissue is critical for the exhibition of a therapeutic effect. To aid this process, the incorporation of a chemokine receptor transgene into the CAR design was assessed. In a model of AML, Jamali and colleagues, generated transgenically augmented anti-CD19 CAR-NK cells (TRACKs) expressing the chemokine receptor CXCR4, which is implicated in the retention of NK cells in the bone marrow niche (149). Improved migration and superior lysis of target cells was demonstrated *in vitro*. The strategy was also applied in a solid tumor setting, where expression of CXCR4 by EGFRvIII CAR-NK cells induced specific chemotaxis towards the CXCL12/SDF-1α positive glioblastoma cell line U87-MG (150).

In the occasion that one of the tumor-associated antigens overexpressed in the tumor is a death receptor, transduction of CAR-NK cells with its respective apoptosis-inducing ligand could effectively redirect CAR-mediated antitumor responses towards the cancer cells. The approach was assessed by Lee Ye and collaborators in a pancreatic ductal adenocarcinoma model, where TRAIL transgene was cloned in the FRα-specific CAR vector (151). The generated CAR-NK-92 cells demonstrated enhanced and targeted cytotoxicity.

### CRISPR/Cas9-Mediated Gene Editing

CRISPR technology is a powerful gene editing tool that has been increasingly used in cellular immunotherapy. CRISPR/Cas9 is successfully used to insert the CAR transgene into specific loci of the effector cell genome with high precision (152). Apart from that, the technology is extensively studied in the context of increasing the functionality and persistence of CAR-modified cells. Such strategy involves the knocking in of genes associated with effector cell activation, and/or the knocking out (KO) of inhibitory genes. In a study on EGFRvIII CAR-T cells, the authors used the CRISPR/Cas9 system to specifically disrupt the PD-1 gene without causing further alterations to the CAR-T phenotype (153). This resulted in the *in vitro* inhibition of the glioblastoma cell growth. Similarly, CRISPR-mediated KO of the endogenous TGF-β receptor II (TGFBR2) in CAR-T cells unleased immunosuppressive breaks and reduced the CAR-T exhaustion (154). In NK cells, blockade of NKG2A expression resulted in highly functional NK cells that overcame NKG2A-mediated inhibition (155). Although the last study used specifically designed protein expression blockers, rather than the CRISPR-technology, it is believed that both methods could be applied for the generation of NKG2A^null^ NK cells.

An alternative way of using the CRIPSR/Cas9 system is enabling the application of an approach in a particular setting. For example, CD38 is a validated target for MM therapy, as it is overexpressed on the malignant cells. However, CD38 is also highly expressed by effector immune cell types, such as the NK cells (156). Because of that, antiCD38 CAR-NK therapy is characterized by effector cell fratricide shortly after the CAR is expressed. In a study of antiCD38 CAR-NK cells, CRISPR/Cas9 KO of CD38 on the NK cells provided a practical solution to avoid fratricide while maintaining their functionality and cytotoxic potential (65).

### Enhancing NK Cell Metabolism

The metabolic state of the CAR-NK cells in the hostile environment of the TME affects their functional fate to a great extent. For example, limiting glycolysis or oxidative phosphorylation (OXPHOS) is known to impair the production of cytokines, such as IFNγ and Granzyme B in NK cells (157, 158). Efforts are made to elevate NK cell metabolism in the TME using gene editing techniques. Mammalian target or rapamycin complex 1 (mTORC1) is a key regulator of NK cell development and effector responses, activated upon cytokine stimulation (e.g IL-15). A method to maintain mTORC1 within the TME is *via* deletion of the cytokine-inducible SH2-containing protein (CIS) (159). This results in increased JAK/STAT and mTORC1 signaling after IL-15 stimulation, and consequently improves metabolic fitness, cytotoxicity and *in vivo* persistence (160, 161). However, caution should be taken during long exposure of the edited NK cell to IL-15, as it may lead to opposite results, such as NK cell exhaustion and reduced cytotoxicity (24). Another pathway that could be targeted is the cMyc signaling. Adequate levels of cMyc protein are of vital importance for sustaining elevated rates of glycolysis and OXPHOS in NK cells (162). Nevertheless, within TME it is observed a rapid loss of cMyc expression. A strategy to sustain the levels of cMyc is by targeting its degradation pathway mediated by the kinase glycogen synthase kinase-3 (GSK3). Indeed, GSK3 inhibitors are found to restore NK cell cytotoxicity and enhance IFNγ and TNFα production in *in vivo* models of AML and ovarian cancer (163, 164). Alternatively, expression of cMyc protein is rescued by increasing the availability of glutamine. Suppression of glutamine metabolism in tumors can be achieved by treatment with the compound JHU083 (165). Last but not least, the potential benefit of the hypoxia-inducible factor 1α (HIF1α) deletion is being discussed, although the complexity of its regulation requires further investigation (166).

### Increasing CAR-NK Cell Homing and Tumor Infiltration

With the advances of CAR technology and the discovery of alternative NK cell sources, many of the initial obstacles that CAR-NK therapy faces were addressed. However, there are still opportunities for further improvements. As we have previously discussed, NK cell homing is crucial for the success of immunotherapy. Reaching the appropriate effector to target cell ratio within the malignant site has proven a challenge, especially with regards to solid tumors (167). Independent studies have provided evidence that 1) pre-conditioning of patient with lymphodepletion (168), 2) complement cytokine support (76), 3) pharmacological intervention (169, 170), 4) reduction of immunosuppression in TME (171–173) and 5) expression of chemokine receptors by effector cells (174), among others, have a positive effect. Combining different strategies into a multi-dimensional novel approach appears to be the best chance of improving the baseline trafficking and infiltration.

## CAR-NK Cells in Combinational Approaches

### Modification of Targeted Antigen Expression

Pharmaceutical intervention can counteract antigen escape by increasing or maintaining the expression of the targeted antigen. The drugs used for this application are mediating epigenetic modulations, post-translational modulations or inhibit antigen cleavage from the cell surface (175). An example is the use of all-trans retinoic acid (ATRA), an anti-leukemic agent known to enhance the expression of CD38 and folate receptor β (FRβ), to increase the potency of anti-CD38 (176) or anti-FRβ (177) CAR-T therapy respectively. Similarly, treatment with histone deacetylase inhibitors, such as valproic acid, causes upregulation of the NKG2DL MICA/B and ULBP2 from the tumor cells (178). This effect was successfully harnessed in a study of NK cell-mediated lysis of hepatocellular carcinoma cells (179). It could additionally be exploited, however, by the NKG2D-based CAR modified T (180) and NK cells (124, 181). Other examples of drug CAR therapy combinations reported in literature are: 1) adenosine 2a receptor antagonists and mesothelin CARs (182), 2) DNA methyltransferase inhibitors (e.g decitabine) and mucin 1 CARs (183), 3) enhancer of Zeste homolog 2 (e.g GSK126 and tazemetostat) and GD2 CARs (184), protein kinase C modulators (e.g bryostatin-1) and CD22 CARs (185), and 4) γ-secretase inhibitors and BCMA CARs (186).

### Anti-Angiogenic Agents

The efficacy of CAR-NK therapy against solid tumors is limited. To tackle problems associated with insufficient migration of the modified cells to the solid tumor, several studies have investigated the synergistic effect of anti-angiogenic agents with CAR-NK cell infusion. Zhang and colleagues investigated the combination of regorafenib and EpCAM-specific CAR-NK-92 cells in a mouse model of human colorectal cancer xenografts and found the combination to have superior antitumor response compared to each monotherapy (169). Another study by Wu et al. combined apatinib with anti-HER-2 CAR-NK-92 cells and assessed the efficacy against gastric cancer xenografts (170). The strategy achieved improved CAR-NK cell infiltration into the larger tumor xenografts and resulted in better tumor growth suppression (169, 170).

### Immune Checkpoint Inhibition

Checkpoint inhibition and adoptive cell transfer have revolutionized modern cancer treatment. The combination of these individually successful immunotherapeutic approaches has been assessed. Specifically, blockade of the immune checkpoint CD73 enhanced the cytotoxicity of NKG2D-targeting CAR-NK-92 cells in CD73^+^ human lung cancer xenograft models (128). Furthermore, the administration of an anti-PD-1 monoclonal antibody together with anti-HER2 CAR-NK-92 cells for the treatment of glioblastoma has been reported in an abstract by Strassheimer et al. (187).

### Therapeutic Antibodies

The combination of adoptive NK cell transfer with therapeutic monoclonal antibodies (mAbs) is a promising therapeutic approach, due to the innate ability of NK cells to induce ADCC *via* its FcγRIII receptor CD16 (188). The efficacy of this combination is dependent on the CD16 polymorphisms, as well as the affinity of the mAb to the CD16 receptor (189). Engineered Fc receptors, with optimized affinity and enhanced durability within the *in vivo* environment have emerged the last years in an effort to increase the efficacy of cell and antibody therapy combination. Indeed, iPSC-derived NK cells expressing a high affinity non-cleavable CD16-construct (hnCD16-iNK cells) showed enhanced ADCC-mediated effector functions against target cells coated with an anti-CD20 mAb (190). To our knowledge, CAR-NK cells have not been combined with mAbs to this day in a peer-reviewed journal. However, a relevant approach was proposed by Goodridge and colleagues in an abstract form, where iPSC-NK cells co-transduced with hnCD16 and antiCD19-CAR constructs showed promising results in combination with Rituximab (191).

A similar approach was described in a study reporting the combination of anti-TF CAR-NK cell therapy with chimeric antibody-like homodimer immunoconjugates that also target TF, called ICON and L-ICON, in triple-negative breast cancer (192). The combination was assessed *in vitro* showing enhanced cytotoxicity deriving both from the CAR and the ADCC response, compared to the individual treatments.

### Radiotherapy

Radiotherapy is a commonly used regime for the treatment of cancer, particularly in solid tumor malignancies. Radiation introduces cell damage to the tumor and the adjacent cells which generates neoantigens or induces stress ligand upregulation (193). The accumulation of activating ligands triggers the immune system and facilitates cancer cell recognition. For this reason, radiotherapy and immunotherapy have been assessed in a combinational approach with promising results (194). In 2020, Kim et al. reported their findings from the synergy of radiotherapy and ACT of *ex vivo* activated NK cells in a human triple-negative breast cancer xenograft model (195). The combination treatment showed enhanced NK cell tumor infiltration, reduced tumor burden, prolonged NK cell retention to the tumor site and suppression of metastasis. CAR-based therapy and radiotherapy have been assessed together only in the context of CAR-T therapy for glioblastoma treatment. In this study, NKG2D-specific CAR-T cells were combined with radiation therapy due to the upregulation of NKG2DL occurring post-radiation (196). The study showed increased CAR-T cell activation and improved outcomes in terms of survival and tumor control. Taken together, the combination of CAR-NK cells and radiotherapy is worth exploring.

### Oncolytic Virotherapy

Oncolytic virotherapy is a fast-developing field within the cancer immunotherapy, attracting particular interest the last two decades. The field is based on the notion of utilizing viruses to selectively replicate within malignant cells, leading to target cell lysis while normal cells remain unaffected (197). Indeed, oncolytic viruses (OVs) have shown impressive cancer cell elimination in murine models, without causing severe side effects in multiple studies (198–200). There are currently three OVs that have received governmental regulatory approval: 1) the herpes simplex virus T-VEC (Imlygic), approved in USA and EU, 2) the adenovirus H101 (Oncocrine), approved in China, and 3) the Rigvir enterovirus, approved in Latvia, Armenia, Georgia and Uzbekistan (201). OVs and CAR therapy have been successfully combined, although mainly in the context of CAR-T cells. The combinational approaches have the form of either sequential treatment courses of OV and CAR therapy, or CAR cells are used as the vessels to deliver OVs to the tumor site (202). There are many advantages of combining two immunotherapies with different mechanism of action. These include better tumor infiltration of the CAR cells following the initial direct tumor cell lysis by the OVs, upregulation of stress markers from the OV-infected cells leading to enhanced effector cell persistence, proliferation and functionality and additional antitumor activity in the case the CAR cells become anergic in the TME (203). CAR-NK cells and OVs have also been studied together. More specifically, in a mouse model of breast cancer brain metastasis, sequential intratumoral administration of OVs (herpes simplex virus) and anti-EGFR CAR-NK-92 cells resulted in better tumor control and prolonged survival, compared to the effects of the individual treatments (204). Moreover, in the *in vitro* experiments of the same study, CAR-NK cells displayed higher cytolytic activity and cytokine release after co-culture with breast cancer cell lines. Additional evidence on the potential of such combinational approach was given by a recently published study on glioblastoma, where OVs expressing the IL-15/IL-15Rα complex (OV-IL15C) were combined with off-the-shelf EGFR CAR-NK cells demonstrating strong antitumor response (205).

### Recombinant Viruses

The combination of recombinant viruses with CAR-NK cells has been reported in a preliminary abstract format. Specifically, HER2-specific AAV-mediated gene transfer of a PD-1 inhibitor together with local administration of anti-HER2 CAR-NK-92 cells has been suggested for the treatment glioblastoma (206).

### CAR-NK Cells as Drug Carriers

An interesting approach on limiting the insufficient delivery of nanoparticle-based drug formulations was suggested by Siegler et al, which involved the use of CAR-NK cells as carriers (207). The researchers used Abraxane, an FDA-approved nanoparticle-based formulation of the chemotherapeutic agent paclitaxel, to load multilamellar liposomal vesicles, which they then cross-linked to the CAR-NK cell surface. The combinatorial approach was assessed *in vitro* and *in vivo* against HER2^+^ and CD19^+^ cancers where enhanced targeted cytotoxicity was observed.

### CAR-NK Cell-Derived Extracellular Vesicles (EVs)

Although less of a combinational approach and more of an unexplored field, CAR-NK cell-derived EVs are worth mentioning. EVs are membranous vesicles secreted by multiple cell types. They enclose proteins, lipids, nucleic acids and other cytoplasmic components of the parent cell and, upon uptake, they mediate biological effects, phenotypic changes and cell-to-cell communications (208). EVs can be categorized into microvesicles, exosomes and apoptotic bodies, depending on their generation mechanism and size. Of particular interest are the exosomes. These are typically 30-150nm in diameter and originate from the endosomal cell compartment. Studies on the content of the NK-cell derived exosomes (NK-exos) revealed that they are loaded with perforin, granzymes, DNAM-1, IFNγ, and other functional molecules, which provides reason for their exploration in cancer therapy (209, 210). Indeed, exosomes derived from the NK cell line NK-92 were found to exert potent antitumor activity in *in vitro* and *in vivo* studies of aggressive melanoma (211). Importantly, NK-exos can be modified, either directly [e.g *via* electroporation (212)] or by the genetic manipulation of the parent cells (213). The latter approach has been extended into the CAR-T field, offering a new and potentially ‘off-the-shelf’ treatment option. More specifically, primary T cells were lentivirally transduced to express a 2^nd^ generation CAR construct (214). The deriving exosomes were analyzed for their content, where it was found that apart from the cytotoxic molecules, the CAR protein was also present. In contrast, the PD-1 receptor was not detected. The cytolytic potential of the exosomes was assessed against relevant cancer cell lines, showing targeted cancer cell death in a concentration-dependent manner. Taking everything into consideration, we believe that the CAR-NK derived exosomes is also an immunotherapeutic platform worthy of exploring.

## CAR-NK Cells in Preclinical Cancer Research

### Hematological Malignancies

CAR-NK-based therapies have been extensively studied for the treatment of hematological malignancies, showing a clear *in vitro* and *in vivo* advantage of CAR expressing NK cells over control NK (41, 46, 63, 149, 160, 190, 215–217). CD19 targeting has been in the epicenter of this research, following the FDA approval of the antiCD19 CAR-T products Yescarta and Kymriah. Overall, multiple studies demonstrated that CAR-NK cells were efficient in eradiating CD19^+^ targets (41, 46, 63, 149, 160, 215–217). Regarding the nature of the assessed CAR constructs, it was shown that 2^nd^ generation anti-CD19 CAR-NK cells containing CD3ζ and CD28 costimulatory domains outperformed 1^st^ generation CARs, whereas 2^nd^ and 3^rd^ generation CAR constructs did not seem to confer substantial differences in NK cell functionality (215, 217). Investigations on the optimal NK cell source were also conducted, where it was suggested that primary NK sources might be a better option than antiCD19 CAR-NK-92 cells (218, 219). In a comparison of CAR PB-NK versus CAR UCB-NK expressing the same antiCD19 CAR construct, it was shown that the former was better in eliminating CD19+ target cells in an effector to target ratio of 1:1 (46). However, the latter could be obtained at higher numbers with less inter-donor variability and stimulation with IL-2 and IL-15 improved more their functionality compared to CAR PB-NK cells. Naturally, the question of whether anti-CD19 CAR-T or CAR-NK cells had better efficacy was addressed. A study comparing CD19-targeting CAR T cell and CAR PB-NK cell anti-leukemia responses *in vivo* demonstrated prolonged survival and reduced adverse effects in mice treated with the CAR-NK cell product, highlighting the potential of CAR-NK therapy in CD19^+^ malignancies (216).

Other studies have evaluated the preclinical efficacy of CAR-NK cell products for the treatment of hematological malignancies targeting CD5, CD20, CD38, FLT3 or B7H7. All these CAR-NK cell products showed enhanced antitumor responses against target cells expressing the respective antigen (65, 125, 131, 138, 190, 220).

### Solid Tumors

CAR-NK therapy has been evaluated as a therapeutic option of various solid tumors, targeting different antigens for each application. More specifically, CAR-NK cell therapy has been assessed in ovarian cancer (NKG2D ligands (NKG2DL), PSMA, FRα, CD24, HER2 or mesothelin) (113, 124, 221–224), glioblastoma (NKG2DL, EGFRvIII and ErbB2) (128, 225, 226), colorectal cancer (NKG2DL and EpCAM)124,187, prostate cancer (PSMA and NKG2DL) (128, 227), hepatocellular carcinomas (c-MET, GPC3 or CD147) (70, 127, 228), pancreatic cancer (mesothelin and FRα) (151, 229, 230), high-risk myosarcoma (ErbB2) (231), gastric cancer (HER2) (170), breast cancer (ErbB2, EGFR and TF) (192, 232, 233), head and neck cancer (PD-L1) (234, 235), neuroblastomas and melanoma (GD2) (236) and lung cancer (NKG2DL and EGFR) (128, 190). Overall, these preclinical studies showed superior antitumor responses *in vitro* and/or *in vivo* compared to non-transduced or control NK cells. However, solid tumors pose additional challenges for CAR-NK cell efficacy, namely intra-tumor infiltration, tumor trafficking, immunosuppressive microenvironment, among others (237). Strategies to overcome these issues and enhance CAR-NK cell functionality against solid tumors have been previously discussed.

## CAR-NK Cells Beyond Cancer Therapy

### Infectious Diseases

A number of studies have explored the potential of CAR-NK therapy for the treatment of infectious diseases, such as AIDS (acquired immunodeficiency syndrome) and COVID-19 (coronavirus disease). Regarding AIDS, a universal CAR-NK cell product was designed to recognize 2,4-dinitrophenyl (DNP)-tagged antibodies that target the gp160 glycoprotein expressed on the HIV-infected cells (238). The study demonstrated effective degranulation against gp160+ cells, as well as killing of HIV-infected primary CD4+ T cells. Although a comparison between DNP CAR-NK cells and anti-gp-160 CAR-NK cells showed the first to be less cytotoxicity, the versatility of the approach and the ability to target multiple variants/isoforms of the HIV gp160 glycoprotein depending on the DNP-tagged antibodies used is an advantage. Therefore, considering the high mutational rate of HIV, the universal DNP CAR-NK cell product poses a very attractive and potentially effective strategy to treat AIDS.

COVID-19 is an infectious disease caused by the recently emerged SARS-CoV-2 virus (239). The virus can cause severe acute respiratory syndrome, for which no effective treatment exists at the moment. Independent studies have explored the potential of ‘off-the-shelf’ CAR-NK cell therapy in this setting, although only one of them is published in a peer-reviewed paper. In that study, Ma and colleagues generated a CAR-NK-92 cell product using the scFv domain of the neutralizing antibody S309, which recognizes a highly conserved region of the virus’ spike glycoprotein (240). S309 CAR-NK-92 cells showed increased degranulation and cytotoxicity *in vitro*, while targeting four different variants of the spike protein. Other studies generated similar spike protein-targeting constructs displaying promising results (241).

### Autoimmune Diseases

The incapability of follicular helper CD4+ T cells (T_FH_) to prevent aberrant immune responses is associated with the development of several autoimmune diseases (242–244). Current therapeutic strategies are insufficient in providing a permanent solution and are additionally causing serious side effects. CAR-NK therapy holds potential, as it can confer targeted elimination of the pathological immune cells in the autoimmune milieu. For their approach, Reighard and colleagues targeted PD-1, a marker moderately expressed on physiological cells, but overexpressed on T_FH_ cells (245). They generated antiPD-1 CAR-NK-92 cells and reported cytotoxicity against PD-1^high^ but not PD-1^low^ cells *in vitro* studies. The results were further validated in an NSG lupus-like mouse model. Autoantibodies have been described in many other autoimmune diseases, such as inflammatory bowel disease and rheumatoid arthritis, where the potential of CAR-NK cell therapy could be investigated.

## Clinical Studies on CAR-NK Cells

To our knowledge, only a few studies evaluating the clinical efficacy of CAR-NK cell for the treatment of hematological malignancies and solid tumors have been published to date. The clinical studies evaluated NK-92, PB-NK and UCB-based CAR-NK cell products.

### NK-92-Based CAR-NK Therapy

A phase I clinical study in patients with AML evaluated the safety of (60) Co-irradiated antiCD33 CAR-NK-92 cells. The cells were transduced with a lentiviral vector encoding for a 3^rd^ generation CAR with CD28, 4-1BB and CD3ζ co-stimulatory domains (246). All three of the enrolled patients were recruited after experiencing relapse from at least one chemotherapeutic regimen. They displayed up to 37,5% blasts in the BM, of which 20.4 to 99.9% were CD33^+^. Overall, the treatment was found well-tolerated by all patients and the maximum tolerable dose was not reached even with the dose of 50 billion cells. The first two patients were diagnosed with grade I cytokine release syndrome (CRS). To evaluate the response to the treatment, bone marrow aspirates were collected 1-4 months post-infusion. Only one of the patients achieved objective response (OR), but shortly relapsed and all three patients eventually reached a concentration of blasts of at least 75%. Analysis of CD33 positivity in two of the patients revealed 49% and 94,6% CD33+ blasts. The limited efficacy was hypothesized to be primarily due to the decreased cytotoxic potency that the CAR-NK-92 cells had after the irradiation step, as well as due to the insufficient phenotypic evaluation of the CD33^+/high^ AML populations. As a solution, the researchers proposed treatment with non-irradiated CAR-NK-92 cells engineered with a suicide gene. This could allow a better control of the lifespan and proliferation of the NK-92 lymphoma cell line in the patients whilst maintaining high viability and cytotoxicity. Further improvements would be the optimization of the CAR construct towards recognizing different CD33 isoforms present in AML patients, and/or the targeting of alternative antigens. Finally, the authors acknowledge the need to elucidate the factors that determine CAR-NK-92 responsiveness in AML in order to better predict the response in patients.

Another study published the results of the clinical evaluation of anti-Robo1 CAR-NK-92 to a patient with pancreatic ductal adenocarcinoma and liver metastasis (NCT03941457) (247). Here, cells were generated with a lentiviral vector carrying a 2^nd^ generation CAR with 4-1BB and CD3ζ co-stimulatory domains. The patient was refractory to chemotherapy and had Robo1^+^ non-operative tumor. The patient was infused with 10 billion anti-Robo1 CAR-NK-92 cells on days 1 and 3, and liver metastasis was treated with percutaneous administration of this CAR product on days 2 and 4. Overall, no substantial treatment-related adverse events were reported. The patient progressed two months after the final infusion, with an overall survival of 8 months.

### Primary Cell-Based CAR-NK Therapy

CAR-transduced UCB- and PB-NK cells have also been investigated in clinical trials. The promising preclinical results obtained with CAR UCB-NK cells co-transduced with IL-15 and iCasp9 genes encouraged the investigation of the clinical efficacy of this therapy (41). Therefore, a phase I/II study was published 2 years later using UCB-NK cells retrovirally transduced with a 2^nd^ generation antiCD19 CAR construct endowed with CD28 and CD3ζ intracellular signaling domains (NCT03056339) (140). In addition, these UCB-NK cells were also transduced with IL-15 gene and iCasp9. Briefly, a single dose of HLA-mismatched antiCD19 CAR UCB-NK cells was administered to 11 relapsed/refractory patients with CD19^+^ lymphomas after undergoing lymphodepleting chemotherapy. These patients already received a median of 4 lines of therapy before. The administered doses ranged from 1 to 100 x 10 (5) cells per kg. The maximum tolerated dose was not reached, and no CRS, neurotoxicity or GvHD was detected. Despite the HLA-mismatch, CAR-NK cells were found at least 12 months after infusion, probably due to the inclusion of IL-15 gene in the engineered cells. With a median follow-up of 13.8 months, the ORR is 73% (8 patients), with 7 patients showing CR and 1 PR.

Last, colorectal cancer patients were treated with anti-NKG2DL CAR-expressing autologous or allogeneic PB-NK cells in an haploidentical setting (125). The product was generated by mRNA electroporation of the CAR gene which contained a single DAP12 co-stimulatory domain. Intraperitoneal infusion reduced EpCAM^+^ cancer cells in two patients, while a third patient showed tumor size reduction four days after the first injection with allogeneic CAR-NK cells. In addition, the third patient showed almost no uptake of fludeoxyglucose by PET/CT imaging after completion of the treatment, an indication of tumor regression. Tumor sites injected with CAR-NK cells demonstrated necrotic lesions, which were not apparent in non-injected tumor sites. Moreover, CAR-NK cell injected tumor regions showed loss of expression of the NKG2D ligands MICA/B, Villin and CDX2 (markers of adenocarcinoma of intestinal origin), supporting the argument of local antitumor effect in the patients.

### Ongoing Clinical Trials

The current ongoing clinical studies evaluating the safety and efficacy of CAR-NK cell therapy in various indications are summarized in **Table 2**. We found that about half of the listed trials are based on the NK-92 cell line. These much-anticipated results are believed to shed light on the potential of NK cell lines as the source of off-the-shelf CAR-NK cell products. The rest of the trials with disclosed information concern mostly PB- and UCB-based CAR-NK cells, while there is also one iPSC-based CAR-NK cell trial (NCT04245722). Being the first registered trial investigating the clinical efficacy and tolerability of this approach, the insights gained from this study could boost further investigations of iPSC-derived CAR-NK cells in the clinical setting.

Even though the applicability of CAR-NK cell therapy in various indications has been proved preclinically, it is clear that the vast majority of the registered clinical trials is focusing on cancer. CD19 remains the most commonly targeted antigen (34% of the trials), while ROBO1 and NKG2DL are being increasingly investigated, counting for about 10% of the listed trials each. It is also worth mentioning that although cell therapy is traditionally used to treat hematological malignancies, there are currently 10 registered trials focusing on solid tumors. Moreover, for the first time, CAR-NK cells are under clinical investigation for the treatment of the pandemic-causing infectious disease COVID-19 (NCT04324996).

As previously mentioned, NK cell-based co-stimulatory domains may induce more potent CAR-NK cell-mediated antitumor responses compared to T cell-based CARs (113, 122–126). The potential of NK cell-based CARs is being increasingly investigated in the clinical setting, counting for 37% of the trials with relative disclosed information. The results of these studies are believed to influence the next generation of CAR-NK therapies.

Lastly, it is worth mentioning that non-CAR genetically engineered NK cell products are also under clinical investigation (e.g., NCT03656705 or NCT04023071). The reports of these results are highly anticipated.

## Non-CAR Genetic Modification of NK Cells

**Figure 1:**
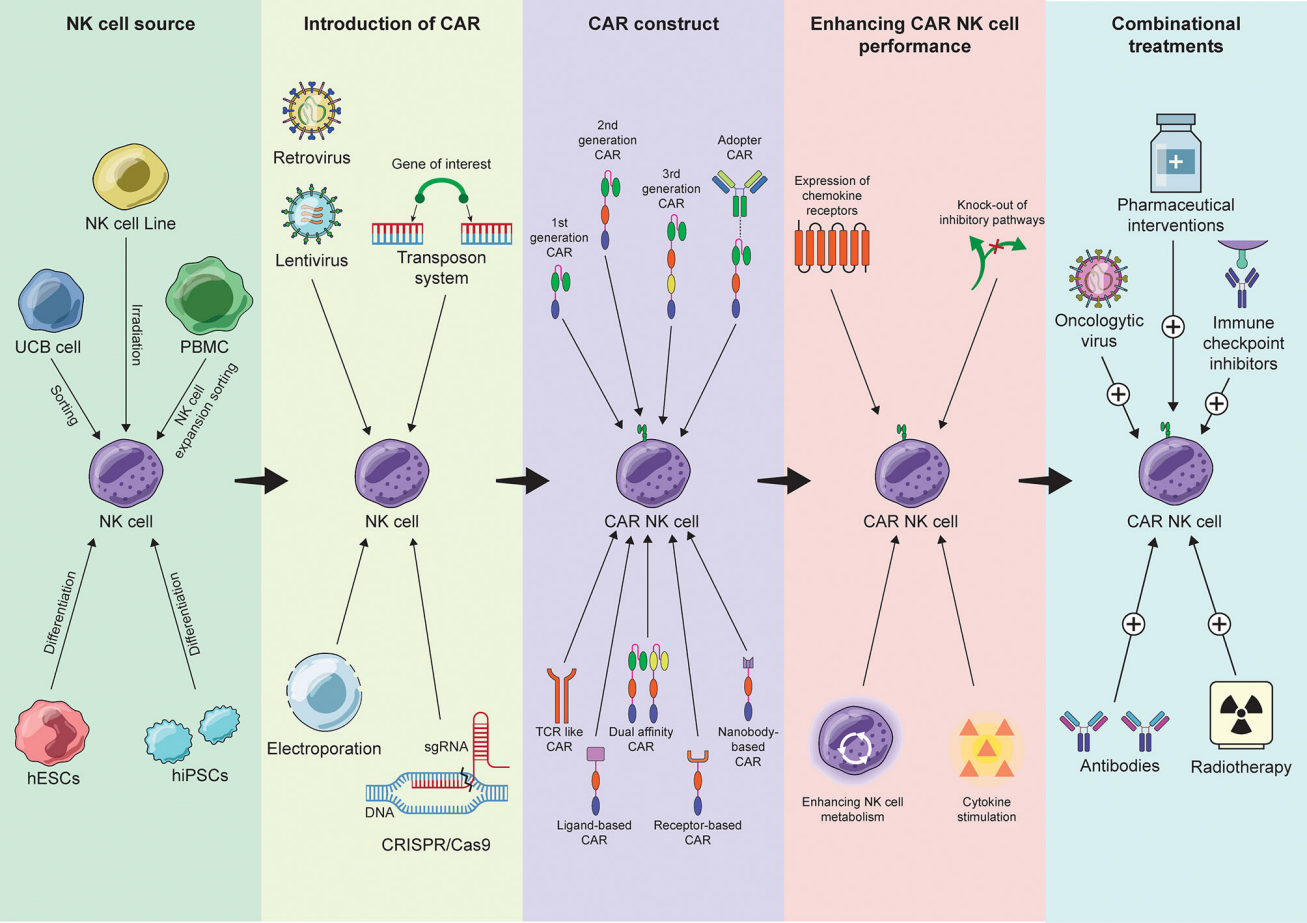
Summary of the recent advances in the process of CAR-NK cell therapy development from NK cell source selection to combinational approaches.

In **Figure 1** we have provided a schematic representation of the different aspects involved in the development of a CAR-NK cell therapy, as well as recent advances of the field. The success story of CAR therapy, as well as the advances in receptor engineering, inspired the development of other constructs for T and NK cell therapy of cancer. Some of the ones that have been applied to NK cells are listed below.

### Dimeric Antigen Receptors (DARs)

Dimeric antigen receptors, or DARs, are a novel category of artificial receptors that share many of the transmembrane and the signal transduction compartments of the CARs (248). While the antigen-targeting domain of the CARs is a scFv domain, however, DARs utilize the whole Fab part of the antibody. This is presumed to increase the stability of the synapse, as well as the targeting specificity. To this day, evidence on the preclinical efficacy of the DARs has only been provided by T cell-based approaches for the treatment of relapsed/refractory multiple myeloma. Nevertheless, the preclinical evaluation of anti-CD38 iPSC-derived DAR-NK cells for the same indication has also been announced.

### Chimeric Switch Receptors (CSRs)

As TME is a major factor dictating the success of an immunotherapy, strategies have been developed to switch the negative effects of immune suppression into positive, using chimeric switch receptors (CSRs). CSRs are cleverly designed to bind to inhibitory ligands on the malignant cells and transmit activating signal instead, thanks to their intracellular signaling domain. The ectodomains of the checkpoint inhibitors PD1 (249), TIGIT (250) and CTLA-4 (251) are particularly attractive and have shown promising results in T cell studies with regards to resistance to immunosuppression and restored effector function (252). Application of the approach to NK cells is gaining popularity the last years. Notably, a study on NK-92 expressing a novel PD1-NKG2D-41BB receptor demonstrated rapid elimination of PD1+ lung cancer target cells in an *in vitro* setting (253). A similar re-targeting approach is the chimeric chemokine receptors (CCRs). Although the potential of CCR-NK cells has yet to be explored, co-expression of chemokine receptors, such as CCR2b, has shown increased migration of CAR-T cells to the site of the malignancy (254).

### T Cell Receptor (TCR)-Expressing NK Cells

The genetic modification of NK cells to express tumor specific T cell receptors (TCRs) has recently been attempted *in vitro*. In contrast to CARs that bind to cell surface antigens, TCRs can recognize antigenic peptides from degraded protein presented on the MHC, and therefore, are theoretically less restricted by the localization of the targeted molecule. An obstacle to the TCR expressing NK cells, however, is the lack of the accessory TCR signaling components that are present in T cells. Taking this into consideration, the engineering of TCR-NK cells becomes more challenging as the presence of the CD3 complex on the cell surface is necessary for the TCR to be functional. Mensali et al, in 2019, provided evidence that TCRs can be successfully expressed on NK-92 cells and that are able to mediate pMHC-specific cytotoxicity (255).

## Concluding Remarks

CAR-NK therapy has given new hope to the patients battling ‘incurable’ diseases and a new platform for researchers to explore the potential of CAR-based cell therapy. Although the challenges regarding the *ex vivo* expansion of the cells, *in vivo* persistence and insufficient cell trafficking remain, recent advances in cell and molecular biology provide viable solutions. Furthermore, CAR-NK cells are proven versatile and customizable, which expands their applicability to diseases beyond cancer. Looking into the future, next generation CAR-NK therapy is incorporating more and more state-of-the-art technology, adapting from the discoveries of CAR-T research, but also harnessing the unique features of NK cells. Taken together, CAR-NK therapy is believed to play an even greater role in the clinics in the forthcoming years, by providing efficient and safe off-the-shelf products.

## Author Contributions

MK, MV-M, AL, and EA contributed equally for this review. All authors contributed to the article and approved the submitted version.

## Conflict of Interest

EA is a founder of Vycellix and XNK and he is a consultant for Vycellix.

The remaining authors declare that the research was conducted in the absence of any commercial or financial relationships that could be construed as a potential conflict of interest.

## Publisher’s Note

All claims expressed in this article are solely those of the authors and do not necessarily represent those of their affiliated organizations, or those of the publisher, the editors and the reviewers. Any product that may be evaluated in this article, or claim that may be made by its manufacturer, is not guaranteed or endorsed by the publisher.
